# Association Between COVID-19 and Orthostatic Intolerance in Children: A Retrospective Study

**DOI:** 10.7759/cureus.74857

**Published:** 2024-11-30

**Authors:** Bahram Kakavand, Safia Centner, Aliya Centner

**Affiliations:** 1 Pediatric Cardiology, Nemours Children's Health System, Orlando, USA; 2 Medicine, University of Central Florida College of Medicine, Orlando, USA

**Keywords:** covid-19, covid-19 vaccine, long-covid, orthostatic intolerance, postural orthostatic tachycardia syndrome (pots), pots covid-19, pots covid-19 vaccine

## Abstract

Introduction

SARS-CoV-2 infection (COVID-19) and the COVID-19 vaccine have been linked to the development of persistent symptoms, including orthostatic intolerance (OI) and postural orthostatic tachycardia syndrome (POTS), in both children and adults. POTS is characterized by excessive tachycardia and other symptoms upon standing, significantly impacting quality of life. This study aims to evaluate the clinical and laboratory findings in pediatric patients with post-COVID-19 or post-COVID-19 vaccine OI and POTS.

Methods

This retrospective chart review included pediatric patients under 18 years of age with chronic dizziness or syncope following COVID-19 infection or vaccination. Autonomic studies, including the tilt table test, Valsalva maneuver, deep breathing test, and Quantitative Sudomotor Axon Reflex Test (QSART), were conducted to assess autonomic function. Data on clinical symptoms, autonomic testing results, and sweat production were collected and analyzed.

Results

In total, 16 patients (mean age 15 ± 3 years) were included in the study, with 14 patients developing symptoms post-COVID-19 infection and two post-vaccination. Ten patients (62.5%) met the criteria for POTS, with an average delta heart rate of 49 bpm on tilt table testing. Seven patients (43.75%) showed abnormal results on the Valsalva maneuver, and 50% (four patients) of those who underwent QSART demonstrated small fiber neuropathy. The mean standing norepinephrine level was 520 picograms per milliliter (pg/mL), with some patients showing markedly elevated levels.

Conclusion

This study highlights the growing incidence of POTS and other forms of OI in pediatric patients following COVID-19 infection and vaccination, supporting the link between COVID-19 and autonomic dysfunction in children. Our findings contribute to the growing body of literature on Long-COVID and emphasize the need for greater awareness, as well as further research into its long-term autonomic effects.

## Introduction

SARS-CoV-2 infection (COVID-19) has been associated with persistent symptoms or implicated in the development of new symptoms after recovery from the initial acute phase [1,2]. The term Long-COVID was coined by Dr. Elisa Perego in a tweet in May 2020 to describe ongoing symptoms long after a COVID-19 infection [3]. Common symptoms include fatigue, muscle weakness, sleep disturbance, shortness of breath, anxiety, and cognitive issues, including memory loss, depression, and difficulty with concentration [3]. 

The COVID-19 vaccine can also have long-term adverse effects [4]. Per the Centers for Disease Control (CDC) report, the COVID-19 vaccine has been shown to cause conditions such as myocarditis and pericarditis [5,6]. Studies have shown that other late adverse effects include cutaneous manifestations, arthralgia, neurologic symptoms, ocular symptoms, and metabolic complications [4].

Post-COVID-19 or post-COVID-19 vaccine orthostatic intolerance (OI), including postural orthostatic tachycardia syndrome (POTS), is seen with increasing frequency in children and adults [1,2,7]. OI is characterized by symptoms such as dizziness, palpitations, blurred vision, headache, fatigue, diaphoresis, and weakness upon standing upright [8]. POTS is a chronic autonomic dysfunction disorder that causes similar symptoms, in addition to excessive tachycardia. Patients may also experience presyncopal or syncopal episodes. POTS is defined by a rapid increase of more than 30 beats per minute (bpm) (40 bpm in children) or a heart rate of 120 bpm in adults within 10 minutes of standing [9]. Patients with POTS may experience significant disability and difficulty completing daily tasks [10]. POTS is estimated to affect around 0.2% to 1% of adolescents and adults, with the typical demographics including Caucasian women ages 15 to 45 years [10]. 

There are several tests and techniques that can be used to evaluate autonomic nervous system (ANS) functioning. The tilt table test is a diagnostic procedure used to assess how the body responds to changes in posture. During the test, the patient is strapped to a table that is gradually tilted from a horizontal to a vertical position, while heart rate and blood pressure are continuously monitored to detect abnormalities in autonomic function [11].

The Valsalva maneuver is a test that assesses the function of both sympathetic adrenergic and parasympathetic systems. The patient breathes into a mouthpiece with an open glottis against a pressure of 30-40 millimeters of mercury (mmHg) for 15 seconds. The interpretation of the Valsalva maneuver is split into four phases. In Phase I, blood pressure rises at the onset of expiration due to a sudden increase in intrathoracic and intraabdominal pressure, which compresses the aorta and vena cava. Phase II is divided into early and late stages. In early Phase II, blood pressure steadily drops as a result of sustained compression of the vena cava, leading to reduced venous return, decreased preload, diminished stroke volume, and a subsequent drop in cardiac output. This decline in blood pressure leads to a compensatory increase in heart rate. At the same time, baroreceptors in the carotid and aortic arch are activated, triggering a sympathetic response that increases total peripheral resistance, restoring blood pressure during late Phase II. Phase III is marked by a sudden drop in blood pressure, which occurs when intrathoracic pressure decreases after expiration. Finally, Phase IV involves a blood pressure overshoot, which is influenced by cardiac adrenergic tone [11].

The Valsalva ratio, a key measure of parasympathetic function, is used to analyze heart rate response during the Valsalva maneuver. It is calculated by dividing the maximum heart rate during the maneuver by the minimum heart rate in the 30 seconds following it. This ratio is then compared to age- and gender-specific normative data. A higher Valsalva ratio suggests better parasympathetic function, though it typically declines with age [11].

In the deep breathing test, the subject performs six slow, deep breaths over the course of one minute. The mean delta value is then calculated by measuring the difference between the peak heart rate during inspiration and the peak heart rate during expiration. It is used to evaluate heart rate variability (HRV) as an indicator of parasympathetic function. A normal HRV response to deep breathing is characterized by an increase in heart rate during inhalation and a decrease during exhalation, a phenomenon known as respiratory sinus arrhythmia [11].

The Quantitative Sudomotor Axon Reflex Test (QSART) is a diagnostic procedure used to assess the function of the ANS, specifically its ability to regulate sweating. During the test, a small electrical stimulus is applied to the skin to activate sweat production, and the amount of sweat produced is measured to evaluate the integrity of the postganglionic sudomotor fibers [11].

Elevated levels of standing plasma norepinephrine may be seen in patients with POTS [12]. Normal values are variable and can range from 70 to 1700 pg/mL [13].

Information about Long-COVID OI is often based on a small number of patients, mostly adults, and the long-term outcomes of these patients are not well understood. The objective of this study is to evaluate clinical and laboratory findings in children with post-COVID-19 or post-COVID-19 vaccine OI and POTS.

This article was previously presented as a poster at the 2023 American Autonomic Society Annual Meeting in San Juan, Puerto Rico, on November 16, 2023.

## Materials and methods

This retrospective chart review study was approved by the Nemours Institutional Review Board. In this retrospective analysis, the records of pediatric patients with chronic dizziness, with or without syncope, following a COVID-19 infection or the COVID-19 vaccine were reviewed. Patients underwent autonomic studies, including QSART, and laboratory studies.

The inclusion criteria included children under the age of 18 years, of any gender, with a history of COVID-19 infection and/or COVID-19 vaccine administration, and chronic orthostatic symptoms.

Autonomic studies

In our laboratory, we perform autonomic studies on all patients with chronic orthostatic symptoms. These studies include the Valsalva maneuver, deep breathing test, and tilt table test at 70 degrees for 10 minutes. For the Valsalva maneuver, patients breathe into a mouthpiece against a pressure of 40 mmHg for 15 seconds. Mean blood pressure during late Phase II is compared to baseline mean blood pressure, and pressure recovery time in Phase IV, and Valsalva ratio are calculated. For the deep breathing test, the subject takes six slow, deep breaths in one minute. The mean delta value between peak inspiratory and peak expiratory heart rate is calculated. In our autonomic laboratory, the tilt table test protocol consists of 10 minutes in an upright position at 70 degrees. A Finapres Nova device (Finapres Medical Systems B.V., Amsterdam, the Netherlands) records the entire procedure.

QSART is performed routinely in our laboratory using QSweat (WR Medical Electronics, St. Paul, MN, USA). A 10% acetylcholine solution is instilled into four electrodes placed on the forearm, proximal leg, distal leg, and foot. The solution is iontophoresed at 2 volts (V) for five minutes, followed by a five-minute observation. The area under the curve is used to calculate the sweat volume for each electrode.

## Results

In total, 16 patients (15 female, 1 male) with a mean age of 15 ± 3 years (range, 6-19 years) met the inclusion criteria of the study. In 14 patients, orthostatic symptoms developed during or after COVID-19 infection. In two patients, symptoms occurred after the first or second dose of the COVID-19 vaccine.

The time to symptom development ranged from 0 to 30 days. In seven patients, orthostatic symptoms began concurrently with the infection, and the time to symptom development after the COVID-19 vaccine was one to two days. The time to diagnosis varied between one month and approximately three years.

Ten patients (62.5%) were diagnosed with POTS by autonomic studies, with a delta heart rate of 49 ± 4 bpm on a tilt table test. Six patients (37.5%) did not meet POTS criteria (delta heart rate, 26 ± 4 bpm). These patients were given the diagnosis of chronic OI. Seven patients (43.75%) (including one from the OI group) showed abnormal (flat) late Phase II on the Valsalva maneuver. Table 1 shows the results of the autonomic studies for the entire cohort.

**Table 1:** Mean values of Deep Breathing Test, Valsalva Maneuver, and Tilt Table Test HR, Heart Rate; HRDB, Heart Rate Response to Deep Breathing; I:E, Inspiration:Expiration; bpm, Beats per Minute; mmHg, Millimeters of Mercury

Autonomic Study	Mean ± Standard Deviation
HRDB I:E	1.4 ± 0.07
HRDB Delta Mean HR	26.5 ± 5.4 bpm
Valsalva Ratio	2.1 ± 0.2
Pressure Recovery Time	2.4 ± 0.9 seconds
Baseline Mean Pressure	90 ± 10.0 mmHg
Phase 1 Mean Pressure	108 ± 15.2 mmHg
Late Phase 2 Mean Pressure	86 ± 17.2 mmHg
Phase 4 Mean Pressure	110 ± 17.9 mmHg
Tilt Starting HR	77 ± 11.5 bpm
Tilt Peak HR	118 ± 12.3 bpm
Tilt Delta HR	41 ± 12.9 bpm

Mean standing norepinephrine levels were 520 picograms per milliliter (pg/mL), with three patients (18.75%) exhibiting values above 600 pg/mL (ranging from 800 to 1400 pg/mL). Of the eight patients who underwent QSART, four patients (50%) showed evidence of small fiber neuropathy (one from the OI group).

## Discussion

The SARS-CoV-2 pandemic was a life-altering event for millions around the world, leading to widespread morbidity and long-term sequelae, cumulatively impacting 9%-63% of patients [14]. Long-COVID symptoms include but are not limited to, fatigue, dyspnea, brain fog, post-exertional malaise, muscle pain, cough, sleep disorders, and palpitation. However, the full scope and underlying mechanisms of Long-COVID remain unclear, with its precise definition evolving as more data are gathered. For many patients, Long-COVID results in severe limitations to daily functioning, including physical, emotional, and social challenges, leading to a diminished quality of life [3].

In a statement on behalf of the American Autonomic Society, Raj et al. suggested that the increased number of POTS cases after SARS-CoV-2 infection is related to the very high incidence of this viral infection and not necessarily due to a unique mechanism of COVID-19 [15]. Cantrell et al. reported on 16 adult patients with POTS after confirmed or probable COVID-19 infection [16], with palpitations, fatigue, dyspnea, headache, and syncope/presyncope being the most commonly reported symptoms.

Pediatric experience with Long-COVID and formally diagnosed POTS by autonomic studies is scant. Long-COVID POTS was diagnosed by a bedside standing test in the setting of a rehabilitation clinic [17], where 71% of 92 patients met the criteria for different forms of OI. The growing number of individuals experiencing OI and POTS after COVID-19 raises important questions about the relationship between viral infections, vaccination, and the onset of autonomic dysfunction.

In this study, we presented a series of 16 pediatric patients who developed OI symptoms following SARS-CoV-2 infection or after the COVID-19 vaccine. Of these patients, 62.5% (10 patients) were diagnosed with POTS, and 43.75% (seven patients) showed evidence of cardiac-autonomic dysfunction based on an abnormal Valsalva maneuver. 

Of the patients who underwent QSART, 50% (four patients) demonstrated evidence of small fiber neuropathy. Small fiber neuropathy, characterized by damage to the small nerve fibers that control autonomic functions, is often associated with conditions like diabetes and autoimmune diseases, and its emergence in post-COVID-19 patients suggests a potential neuropathic component to the pathophysiology of Long-COVID POTS. Small fiber neuropathy may contribute to the impaired regulation of blood pressure and heart rate that is commonly observed in these patients [11].

To our knowledge, this is the most detailed description of Long-COVID POTS in children. However, a key question remains: is Long-COVID POTS a distinct entity from other forms of POTS? There is increasing evidence that POTS and other forms of OI can occur after viral infections, suggesting a potential viral trigger for autonomic dysfunction. However, whether the immune response triggered by COVID-19 is unique in its ability to induce POTS, or whether this represents an exacerbation of pre-existing autonomic dysfunction, is still unclear. A larger, prospective study could provide more definitive answers by examining a broader cohort of patients, including those who develop symptoms after different types of viral infections, to identify potential distinguishing features of Long-COVID POTS.

Another important consideration is the role of the COVID-19 vaccine in the development of OI. While the vaccine has been shown to have numerous benefits in preventing severe illness from COVID-19, some patients have reported experiencing symptoms similar to Long-COVID after vaccination, including OI and POTS. In our study, two patients developed orthostatic symptoms following COVID-19 vaccination, and it is crucial to continue monitoring these individuals to assess the long-term effects of the vaccine on autonomic function. Understanding the relationship between the vaccine and the onset of POTS may provide insights into the broader mechanisms at play and help guide future management.

This study also highlights the need for a comprehensive approach to diagnosing and managing OI in the context of Long-COVID. Although autonomic studies, including tilt table tests, Valsalva maneuvers, and QSART, are helpful in identifying autonomic dysfunction, they should be interpreted in the broader context of the patient's clinical presentation. Given the complexity of the ANS and the potential for overlapping symptoms with other conditions, careful differentiation between POTS, other forms of OI, and other potential causes of dizziness and syncope is essential. Multidisciplinary collaboration between cardiologists, neurologists, and autonomic specialists will be crucial in optimizing the care of these patients.

Limitations of this study include the small sample size. In the future, this study could be conducted with a larger sample size and include comparisons in test results between patients with a POTS diagnosis versus those with an OI diagnosis, as well as comparisons between patients who developed symptoms during or after a COVID-19 infection versus those who developed symptoms after the COVID-19 vaccine. More research is needed to understand the long-term outcomes of these patients.

## Conclusions

In conclusion, our study provides valuable insight into the increasing prevalence of OI, including POTS, in pediatric patients following COVID-19 infection or vaccination. The findings highlight the significant impact of Long-COVID on autonomic function in children, with abnormal results in autonomic testing, such as tilt table tests, Valsalva maneuvers, and QSART. Further research is essential to better understand the long-term effects of Long-COVID and develop effective management strategies for affected patients.
